# Flow cytometry datasets consisting of peripheral blood and bone marrow samples for the evaluation of explainable artificial intelligence methods

**DOI:** 10.1016/j.dib.2022.108382

**Published:** 2022-06-17

**Authors:** Michael C. Thrun, Jörg Hoffmann, Maximilian Röhnert, Malte von Bonin, Uta Oelschlägel, Cornelia Brendel, Alfred Ultsch

**Affiliations:** a Databionics, Mathematics and Computer Science, Philipps-Universität Marburg, Hans-Meerwein-Straße 6 D-35032 Marburg, Germany; b J Department of Hematology, Oncology and Immunology, Philipps-University, Baldinger Str., D-35032 Marburg, Germany; c Medizinische Klinik und Poliklinik I Bereich Innere Medizin / Hämatologie und Onkologie, Universitätsklinikum Carl Gustav Carus Dresden, Germany

**Keywords:** Cell populations, Immunophenotyping, Explainable artificial intelligence, Benchmarking, Flow cytometry, Human blood, Human bone marrow, Interpretable machine learning

## Abstract

Three different Flow Cytometry datasets consisting of diagnostic samples of either peripheral blood (pB) or bone marrow (BM) from patients without any sign of bone marrow disease at two different health care centers are provided. In Flow Cytometry, each cell rapidly passes through a laser beam one by one, and two light scatter, and eight surface parameters of more than 100.000 cells are measured per sample of each patient. The technology swiftly characterizes cells of the immune system at the single-cell level based on antigens presented on the cell surface that are targeted by a set of fluorochrome-conjugated antibodies. The first dataset consists of N=14 sample files measured in Marburg and the second dataset of N=44 data files measured in Dresden, of which half are BM samples and half are pB samples. The third dataset contains N=25 healthy bone marrow samples and N=25 leukemia bone marrow samples measured in Marburg.

The data has been scaled to log between zero and six and used to identify cell populations that are simultaneously meaningful to the clinician and relevant to the distinction of pB vs BM, and BM vs leukemia. Explainable artificial intelligence methods should distinguish these samples and provide meaningful explanations for the classification without taking more than several hours to compute their results. The data described in this article are available in Mendeley Data [1].

## Specifications Table

**Table:** 

Subject	Immunology and Hematology
Specific subject area	Immunophenotyping
Type of data	Table
How the data were acquired	*Data were acquired with the following two flow cytometers:* • Dresden: (N=44): BD FACSCanto II™, BD Biosciences (Heidelberg) • Marburg: (N=14 and N=50): Navios™, Beckman Coulter (Krefeld)
Data format	*Raw*
Description of data collection	*The data is readable in any text editor. Each file is the measurement of a patient sample. It consists of more than 100.000 cells (cases or events). For each case, the antigens described below are measured. Both flow cytometers measure forward and side light scatter (FS and SS) and use the same panel of fluorescent antibody clones against the same antigens:* • CD34 FITC (Fluoresceinisothiocyanate) (8G12), • CD13 PE (Phycoerythrin) (L138), • CD7 PerCP-Cy5.5 (Peridinin chlorophyll protein-Cyanine5.5) (M-T701) • CD56 APC (Allophycocyanin) (NCAM16.2) • CD33 PE-Cy7 (Phycoerythrin Cyanine7) (D3HL60.251), • CD117 AlexaFluor750${}^{TM}$ (104D2D1), • HLA-DR Pacific blue${}^{TM}$ (Immu357), • CD45 Krome Orange${}^{TM}$ (J33) Samples are anonymized. Samples are automatically compensated within the instrument settings of the flow cytometer device. Data are scaled in log and have a range between zero and six.
Data source location	Dresden data: Medizinische Klinik und Poliklinik I Bereich Innere Medizin / Hämatologie und Onkologie, Universitätsklinikum Carl Gustav Carus an der Technischen Universität Dresden, Fetscherstraße 74, D-01307 Dresden. Marburg data: Department of Hematology, Oncology, and Immunology, Philipps-University, Baldinger Straße, D-35043 Marburg.
Data accessibility	Repository name: Mendeley DataData identification number: 10.17632/jk4dt6wprv.5 Direct URL to data: https://data.mendeley.com/datasets/jk4dt6wprv/5

## Value of the Data

• For clinicians, the diagnosis of leukemia based on bone marrow samples is a basic task. However, it is an essential question whether bone marrow (BM) samples of patients with hematological diseases are diluted with peripheral blood (pB) in order to assess Minimal Residual Disease status (MRD) in the correct manner (c.f. [2,3]).
• Researchers can use the data in two difficulty levels for three types of assessment of explainable AIs (XAI) or supervised interpretable machine learning methods: performance, interpretability, and processing time.
• Performance (e.g. [4]): The two classes per dataset are balanced, and therefore basic accuracy of the XAI can be evaluated per sample by counting the true positives, false positives, true negatives, and false negatives samples.
• Interpretability (e.g. [5]): Explanations have to be understandable and meaningful to the domain expert (c.f. [6]) within a comprehensible number [7,8].
• Processing time: Due to the large number of cases within a clinical setting, the computations of any algorithm have to finish within an acceptable time (hours) on standard personal computers.
• Researchers can use cases of each sample to benchmark unsupervised machine learning methods (c.f. [9]) because domain experts (i.e., clinicians) distinguish pB samples from BM samples, and leukemia BM samples versus non-leukemia BM samples based on distributions of biological cell populations by looking at two-dimensional scatter plots. As a consequence, clear, straightforward patterns in data that have a biological meaning are visible to the human eye (c.f. [10]).

## 1 Data Description

**Fig 1:**
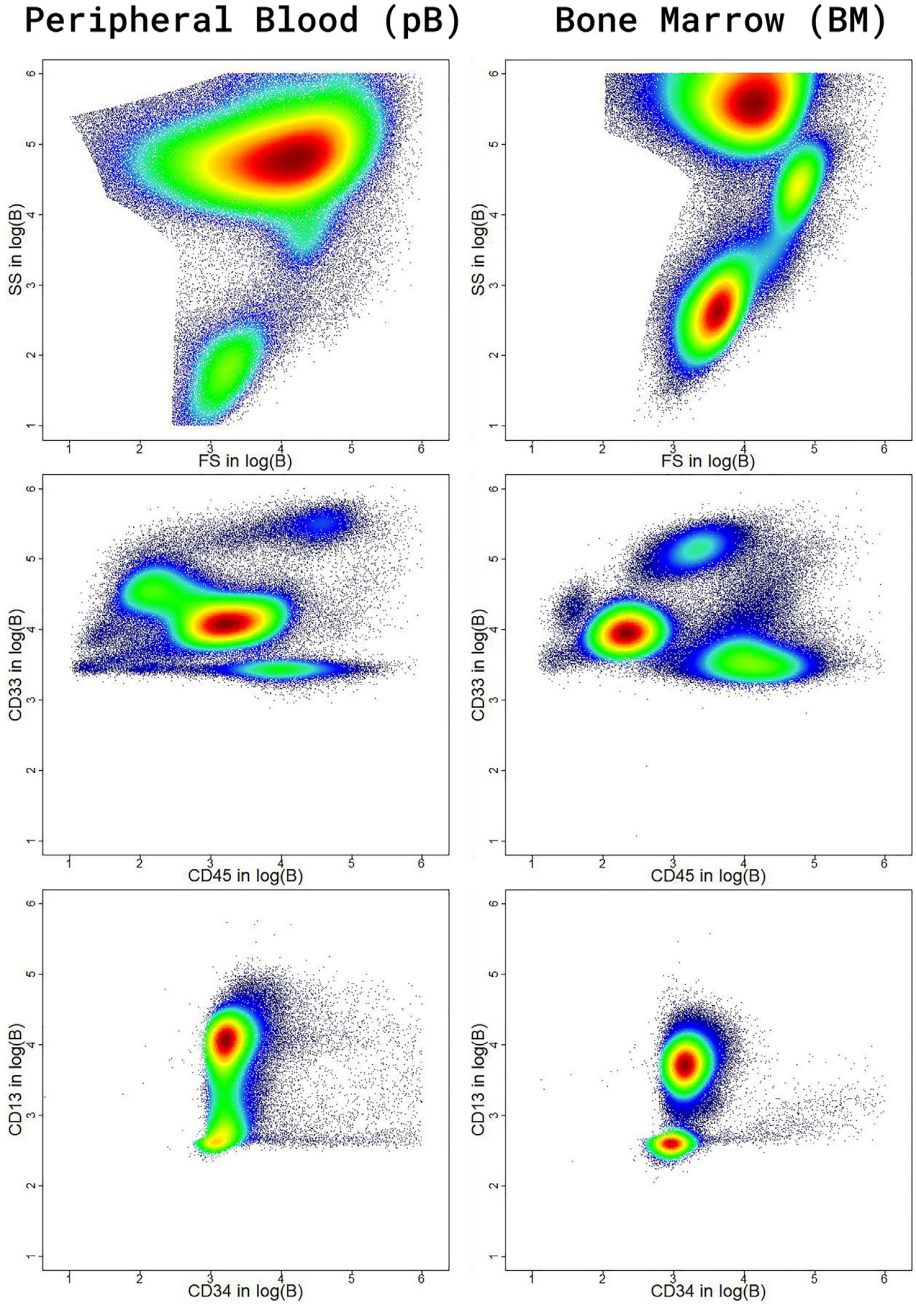
Six two-dimensional scatter-density plots of two sample files of two different patients of the Dresden data. Blue points represent events of cell measurements. The scale of each axis is logarithmic, and a value represents a functional measure of the brightness B of a specific fluorescent antibody clone [11]. With increasing density, a data point's color changes from blue to green, yellow, and then red. The three density plots on the left show ∼800.000 events for a sample file measured from peripheral blood (right - bone marrow).

Data is provided as text files separated by tabular. In addition, there are two overview files per dataset storing the classification information of the sample (pB or BM, see Fig. 1), a unique key, and the file names.

In the subfiles, tab-separated sample files are stored. Each sample file of a patient is anonymized and starts with a comment line indicated by “#” and header lines shown by “%”. The header defines the names of the measured features described above. Thereafter, columns of tab-separated cases with a unique key lieing in the first column follow. Including the key column, there are eleven columns of data per file. Ten columns defining the features are the following: Forward and side light scatter (FS and SS), CD34 FITC (Fluoresceinisothiocyanate) (8G12), CD13 PE (Phycoerythrin) (L138), CD7 PerCP-Cy5.5 (Peridinin chlorophyll protein-Cyanine5.5) (M-T701) CD56 APC (Allophycocyanin) (NCAM16.2), CD33 PE-Cy7 (Phycoerythrin Cyanine7) (D3HL60.251), CD117 AlexaFluor750 (104D2D1), HLA-DR Pacific blue (Immu357), and CD45 Krome Orange (J33).

Each file can be read-in with any text editor, Microsoft Excel, or any programming language. In addition, the R package on GitHub (https://github.com/Mthrun/dbt.DataIO) with extensive documentation is provided for the programming language R in order to read the data. Alternatively, any *.csv reading function can be used.

## 2 Experimental Design, Materials, and Methods

Sample data were acquired with two different flow cytometers: Navios™, Beckman Coulter (Krefeld) for the Marburg data, and BD FACSCanto II™, BD Biosciences (Heidelberg) for the Dresden data, for which ten features were measured. Both measure forward and side light scatter (FS and SS) and use the same panel of fluorescent antibody clones against the same antigens CD34, CD13, CD7, CD56, CD33, CD117, HLA-DR, and CD45. Hence, an event or case consists of these ten features measured as optical characteristics for a cell in the flow cytometer as follows (c.f. [12] for details). Cells pass one by one through the bright center of the consistent and focused laser beams to assess their optical characteristics with the flow cytometer. The light-collecting optics of the laser beams focus on the cells' crossing point to collect fluorescence and dispersed light from cells. The spectral overlap between fluorescent dyes has usually to be accounted for by means of compensation (c.f. [12] for details). The data provided here is compensated using the specific settings defined in the respective flow cytometer. In the case of the datasets provided here, ten features are measured for each event. A value of a feature represents a functional measure of the brightness B of a specific fluorescent antibody clone [11]. The domain expert usually investigates bi-variate visualizations in the logarithmic scale. Exemplary, six density-scatter plots with two features in each are presented in Fig. 1 for two sample files. The left column consisting of three density-scatter plots indicates several cell populations (clusters) for a sample file based on peripheral blood (pB). The right column indicates different cell populations taken from bone marrow (BM). Around 800.000 cells are visualized as data points with colors ranging from blue to red that indicate a rising density. Typically, the domain expert applies several gates consecutively to identify cell populations with biological meaning. For example, In the FS vs. SS density scatter plot (top left) measured for peripheral blood, the domain expert could make “an inclusive gate containing lymphocytes and monocytes to include plasma blasts that are larger in size and more granular than other subsets of B cells” [12]. The plots were generated with the R package "DataVisualizations” available on CRAN [13] using the density estimation SDH [14].

The Marburg dataset consists of n = 7 data files (samples) containing event measures from peripheral blood (BP) and n = 7 data files for bone marrow (BM). The next dataset from Marburg consists of N=25 healthy bone marrow samples and N=25 leukemia bone marrow samples.

The Dresden dataset comprises of n = 22 sample files for peripheral blood and n = 22 samples for bone marrow. Each sample file containes more between 130.000 and 880.000 events. The samples described as BM represent the first ml of aspiration into the first syringe.

Additionally, overall samples of the Marburg data with n=700.000 cases (BM vs. pB) and n=2.500.000 cases (BM vs. leukemia), and the Dresden data set containing n=440.000 cases (events) are provided in separate files. Here, the patient files were randomly subsampled for the training data, and the cases were combined within a single training set per dataset. The three classifications for the cases (events) stored in three separate files were derived from the type of sample (BM, pB, leukemia). The training data was used in the XAI algorithms investigated in [15] to generate populations and corresponding explanations. Testing was performed on the N=14, N=50, and N=44 sample files per patient for which a decision (BM/pB/leukemia) was calculated as a majority vote of the population classifiers.

## Ethics Statements

Data was generated during typical diagnostic procedures and data processing was according to the local ethics committees in Marburg and Dresden (Vote 91/20). Data is anonymized and does not contain any personal information or images of patients.

## CRediT Author Statement

**Michael Thrun, Alfred Ultsch:** Conceptualization, Methodology; **Jörg Hoffmann, Maximilian Röhnert, Malte von Bonin, Uta Oelschlägel, Cornelia Brendel:** Data curation; **Michael Thrun:** Writing – original draft preparation; **Cornelia Brendel:** Writing – review & editing.

## Declaration of Competing Interest

The authors declare that they have no known competing financial interests or personal relationships that could have appeared to influence the work reported in this paper.
